# Construction of an ultrahigh-density genetic linkage map for *Jatropha curcas* L. and identification of QTL for fruit yield

**DOI:** 10.1186/s13068-017-1004-9

**Published:** 2018-01-09

**Authors:** Zhiqiang Xia, Shengkui Zhang, Mingfu Wen, Cheng Lu, Yufang Sun, Meiling Zou, Wenquan Wang

**Affiliations:** 1 The Institute of Tropical Biosciences and Biotechnology, Chinese Academy of Tropical Agriculture Sciences, Haikou, China; 2 Huazhong Agricultural University, Wuhan, China

**Keywords:** *Jatropha curcas*, Ultrahigh-density linkage map, Fruit yield, QTL analysis

## Abstract

**Background:**

As an important biofuel plant, the demand for higher yield *Jatropha curcas* L. is rapidly increasing. However, genetic analysis of *Jatropha* and molecular breeding for higher yield have been hampered by the limited number of molecular markers available.

**Results:**

An ultrahigh-density linkage map for a *Jatropha* mapping population of 153 individuals was constructed and covered 1380.58 cM of the *Jatropha* genome, with average marker density of 0.403 cM. The genetic linkage map consisted of 3422 SNP and indel markers, which clustered into 11 linkage groups. With this map, 13 repeatable QTLs (reQTLs) for fruit yield traits were identified. Ten reQTLs, *qNF*-*1*, *qNF*-*2a*, *qNF*-*2b*, *qNF*-*2c*, *qNF*-*3*, *qNF*-*4*, *qNF*-*6*, *qNF*-*7a*, *qNF*-*7b* and *qNF*-*8,* that control the number of fruits (NF) mapped to LGs 1, 2, 3, 4, 6, 7 and 8, whereas three reQTLs, *qTWF*-*1*, *qTWF*-*2* and *qTWF*-*3,* that control the total weight of fruits (TWF) mapped to LGs 1, 2 and 3, respectively. It is interesting that there are two candidate critical genes, which may regulate *Jatropha* fruit yield. We also identified three pleiotropic reQTL pairs associated with both the NF and TWF traits.

**Conclusion:**

This study is the first to report an ultrahigh-density *Jatropha* genetic linkage map construction, and the markers used in this study showed great potential for QTL mapping. Thirteen fruit-yield reQTLs and two important candidate genes were identified based on this linkage map. This genetic linkage map will be a useful tool for the localization of other economically important QTLs and candidate genes for *Jatropha*.

**Electronic supplementary material:**

The online version of this article (10.1186/s13068-017-1004-9) contains supplementary material, which is available to authorized users.

## Background

As a sustainable and renewable energy source, bioenergy, whose use may reduce dependency on fossil fuels and maintain a safe and healthy environment, has attracted worldwide attention [1, 2]. *Jatropha curcas* L., characterized by drought resistance, low cost of planting, fast growth rate and high oil content, is one of the highest potential energy plants among oil-bearing tree species [3, 4]. Therefore, increasing yield or oil content is the most important breeding objective for *J. curcas* researchers.

*Jatropha curcas* is a native plant, originated in Mexico and Central America. After being introduced into tropical and subtropical areas, it has been widely planted in African and Southeast Asian countries, such as Zimbabwe, China, India, Mauritius, and the Philippines [1, 5]. Already, more than 2 million ha of *J. curcas* are growing in China, mainly distributed in southern and southwestern China, such as Hainan, Yunnan, Guangxi, and Guizhou [1]. *J. curcas* is a diploid species (2*n* = 22), with an estimated genome size of 416 Mb [6, 7]. A previously reported genome sequence of *J. curcas* was 285.9 Mb, with the mean and N50 scaffold lengths of 1.9 and 3.8 kb, respectively [8]. An upgraded *J. curcas* genome sequence was 397 Mb, and the mean and N50 scaffold lengths were 7.6 and 16.0 kb, respectively [9]. Following the upgraded version, the most recent *J. curcas* genome sequence is 320.5 Mb, with the N50 scaffold length being 0.75 Mb [10].

Genetic linkage maps, an important tool for genetic analysis and molecular breeding, have been widely used for identification of genetic loci with agronomic traits such as biological or abiotic stress and yield, which can promote more cost-effective breeding and genetic improvement. The first genetic linkage map, using 93 progeny from an interspecific cross between *J. curcas* and *J. integerrima*, contained 506 markers (216 microsatellite and 290 single nucleotide polymorphism, SNPs), with an average marker density of 2.8 cM [11]. Another genetic linkage map contained 1208 markers, with an average diversity of 1.4 cM per marker [10]. Sun constructed a genetic linkage map with 105 SSR markers [12]. King constructed a genetic linkage map containing 502 markers, in which 399 were unique markers [13]. Subsequently, the same linkage map was moderately improved and contained 587 markers, with a density of 1.2 cM per marker or 1.5 cM per unique locus [14]. The research and development of *J. curcas* is still at a very early stage compared with more established oilseed crops, which have seen significant increases in yield through breeding and agronomy [13]. An available ultrahigh-density genetic linkage map (≤ 1 cM average map density [15–17]) for *J. curcas* has not been reported at present. To rapidly improve fruit yield, *J. curcas* requires numerous informative and well-distributed genome-wide markers for construction of an ultrahigh-density genetic linkage map.

Based on next-generation sequencing (NGS) technology, several high-throughput SNVs discovery methods have been developed including restriction site-associated DNA sequencing (RAD) [18], genotyping by sequencing (GBS) [19], sequence-based genotyping (SBG) [20], and amplified-fragment single nucleotide polymorphism and methylation (AFSM) [21]. As one of the next-generation genetic marker types, the AFSM method has several attractive features for linkage mapping, especially for non-model organisms. AFSM is a simple and rapid method that can be used in SNP and indel discovery by sequencing short genomic regions surrounding restriction sites for a given restriction endonuclease, which produces AFSM markers within the restriction sites or in adjacent sequences that flank the restriction sites. AFSM allows cost-effective whole genome screening for a large number of markers and individuals. AFSM can be used in discovering markers and constructing high-density linkage maps for many plant species and has been successfully employed in cassava [22]. To increase the understanding of the genetic architecture of *J. curcas*, a good available genetic map is required. In this study, we constructed an ultrahigh-density genetic linkage map comprising 3422 SNP and indel markers in *J. curcas* with an average marker density of 0.403 cM. This ultrahigh-density genetic linkage map represents the first ultrahigh-density genetic linkage map of *J. curcas*, and may provide an indispensable and powerful tool for QTL analysis, gene mapping and marker-assisted selection in *J. curcas* breeding. We also identified thirteen reQTLs and two important candidate genes for *J. curcas* fruit-yield traits.

## Results and discussion

### A large-scale SNP and indel discovery in *J. curcas* by AFSM

Illumina HiSeq 2500 sequencing generated a total of 450,514,542 high-quality reads (99.26%) out of a total of 453,860,778 reads. There were 9,202,450 reads for the parents (3,919,586 reads for YN049X and 5,282,864 reads for HN001-31-1) and 441,312,092 for F1 progeny. The F1 progeny had a mean number of 2,922,597 reads, with minimum and maximum number of 44,195 and 27,232,578 reads, respectively.

Here, we used high-throughput Illumina AFSM to detect genome-wide SNPs and indels and to genotype F1 accessions in the *J. curcas* HY population. The 127-bp read length for AFSM tag, as obtained in our study, is longer than that reported in the jute [23] and bitter gourd [24] (approximately 90-bp read length for RAD-tag). For high-density linkage map construction in plants, many F1 or F2 mapping populations were used [24–31]. İpek et al. used an olive F1 population consisting of 123 individuals to construct a linkage map [25]. Balsalobre et al. used 151 full sibs derived from a cross between SP80-3280 and RB835486 sugarcane cultivars to construct a linkage map [26]. An F1 population comprising 153 individuals was used to construct the linkage map in this study, which is similar in number to the individuals used in the sugarcane [26] and larger than the population used in the olive [25].

### An ultrahigh-density genetic linkage map for the HY population in *J. curcas*

Out of 183,650 high-quality markers (165,062 SNPs and 18,588 indels), a total of 73,334 polymorphic markers (with read-depth ≥ 5, SNP base quality ≥ 20, 5% minor allele frequency) were detected in the HY population. We found high polymorphism level between parent lines (38.15%), which was consistent with the previous studies that accessions from China had high polymorphism level [32–34] rather than low [1]. Only markers with a good fit to the expected Mendelian 1:1 or 3:1 segregation ratios were retained. Finally, 6704 markers were used for linkage map construction.

**Fig. 1:**
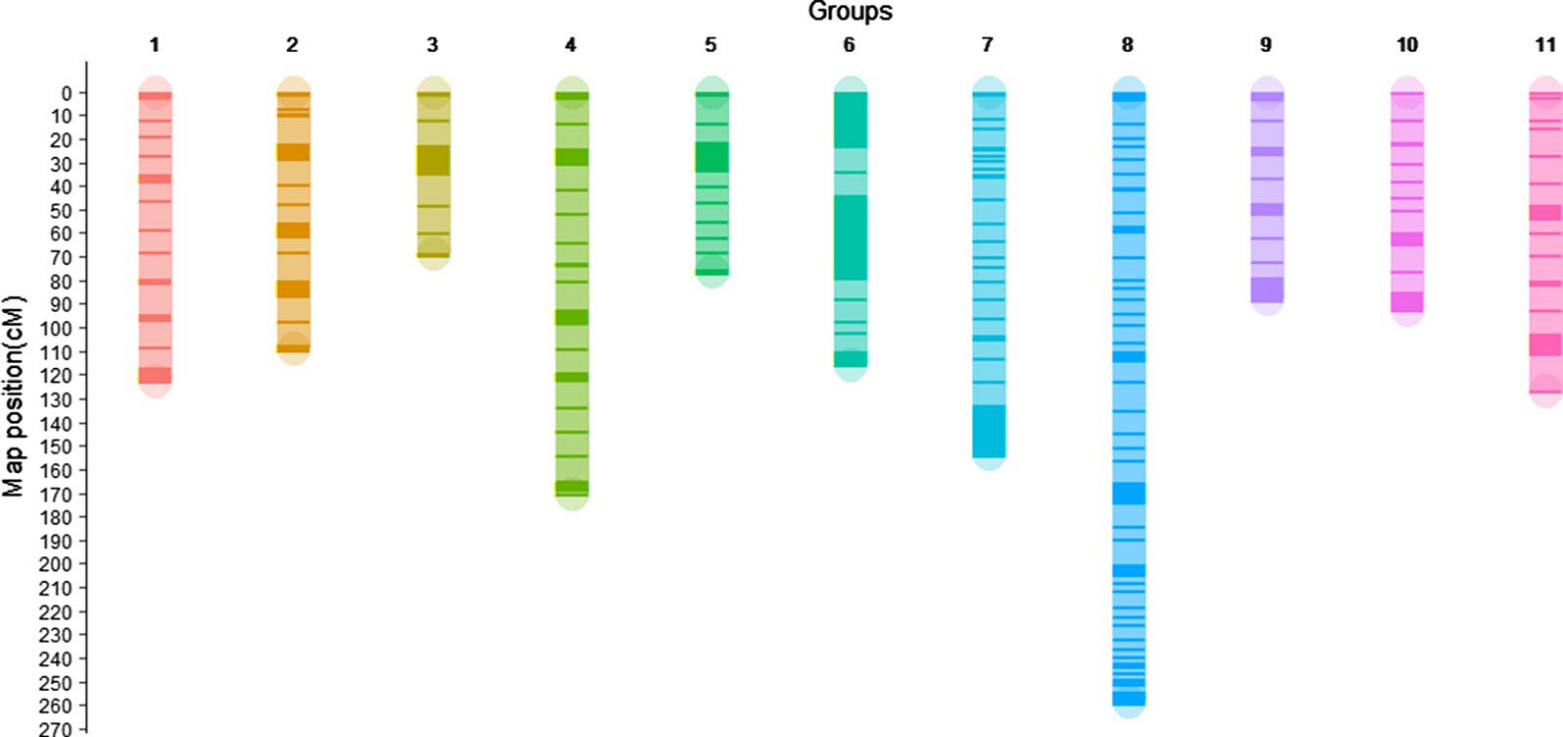
Integrated *Jatropha* genetic map constructed using SNP and indel markers. LGs are the linkage groups. The scale on the left indicates map distance in centimorgans. The darker bands indicate the markers

We constructed the ultrahigh-density genetic linkage map of *J. curcas*, consisting of 3422 SNP and indel markers and covering 1380.58 cM of the genome (Table 1, Fig. 1). The number of markers contained in this linkage map is approximately three times the number of markers in the largest *J. curcas* linkage map previously constructed [10]. These mapped markers were distributed throughout the 11 linkage groups (LGs), which is consistent with the karyotype of *J. curcas* [6]. LG 6 had the highest (664) number of markers, and LG 10 had the lowest (183) number of markers, whereas LG 8 had the longest (258.6 cM) length, and LG 3 had the shortest (68.67 cM) length. The average density was 0.403 cM, which was higher than other available linkage maps of *J. curcas* in the previous studies (2.8 cM, 216 SSR and 290 SNP markers [11]; 1.4 cM, 1208 markers [10]; 1.2 cM, 587 markers [14]) and other ultrahigh-density genetic linkage maps of the jute (0.72 cM, 503 RAD markers) [23], cotton (0.69 cM, 3187 GBS markers) [31], olive (0.98 cM, 3384 GBS markers) [25], and American cranberry (0.93 cM, 1328 GBS markers) [35].

**Table 1:** Distribution and statistics of SNP and indel markers on the linkage groups of the *Jatropha* HY genetic map from the “YN049X” and “HN001-31-1” cross

Linkage groups (LG)	Number of markers	LG length (cM)	Average distance (cM)
LG1	212	122.51	0.578
LG2	373	109.10	0.292
LG3	250	68.67	0.275
LG4	249	170.31	0.684
LG5	286	76.20	0.266
LG6	664	115.35	0.174
LG7	484	153.40	0.317
LG8	291	258.60	0.889
LG9	210	87.86	0.418
LG10	183	92.13	0.503
LG11	220	126.45	0.575
Total	3422	1380.58	0.403

Only using the markers in this study, we were able to anchor a total of 752 scaffolds into our genetic linkage map. The combined length of these scaffolds was 25,411 Mb, which was equivalent to 79.82% of the sequenced genome.

### Recombination events and linkage disequilibrium

Assuming on average two crossovers per chromosome during a single round of meiosis, and 11 chromosomes for each of 153 individuals, the mapping population contains an estimated 3300 total recombination events. Thus, our map of 3422 markers is likely to capture many of the available recombinant genotypes. In fact, our maps invert the traditional relationship between markers and recombination events in high-resolution maps: there are multiple markers within each recombination event rather than vice versa. The recombination hotspots were shown in Additional file 1: Figure S1 and Table S1. The number of recombination hotspots of cross-over detected for 11 LGs ranged from 1 to 3, with a mean of 2.0.

Extent of genome-wide LD was evaluated using all possible pair combinations of 3422 markers genetically mapped on 11 Jatropha LGs genetic maps. We combined the data from 11 LGs to estimate the average extent of LD in *Jatropha*. For these marker pairs, *r*^2^ ranges between 0 and 0.626. Additional file 1: Figure S2 shows the distribution of *r*^2^ values for the marker pairs. Distributions shifted toward the modest *r*^2^ values reflect disequilibrium, due to either chance or evolutionary forces that affect variation across the entire genome. Median *r*^2^ values is ~ 0.058, and the 75th percentile for LD (which we are using to define elevated LD) is ~ 0.176.

The faster LD decayed in population, the more markers are probably needed for QTL trait analysis [36]. We observed faster extended LD decay in LGs of Jatropha (~ 4 cM, Additional file 1: Figure S3) than chickpea (~ 15 cM) [15]. The extent of LD pattern is population dependent as well as expected to vary as a function of recombination frequency along the genome, mating system, and population history including selection. We should note, LD measured in a wild population may be dramatically different from that observed in a breeding population that has been through a genetic bottleneck [37].

The average LD estimate in 11 LGs of Jatropha genome (*r*^2^) was 0.540 (Additional file 1: Table S2). The LG5 of Jatropha genetic maps had highest LD estimates (*r*^2^ = 0.784), while LG8 had lowest LD estimates (*r*^2^ = 0.459). Maximum and minimum proportion of marker pairs showed significant LD (*P* < 0.01) on LG5 (46.800%) and LG4 (17.454%), respectively (Additional file 1: Table S2). We determined the LD decay of 3422 marker pairs by pooling the *r*^2^ estimates across 11 LGs and plotting their average *r*^2^ against the 10 cM equal intervals of genetic distance. A decreasing trend of LD decay (*r*^2^ < 0.3) was observed with increase in the genetic distance (cM) of markers mapped on the LGs (Additional file 1: Figure S3). Remarkably, a rapid LD decay was observed at the genetic distance of 1.6 cM in genomes. A significant LD decay (*r*^2^ < 0.1) was observed near about 4 cM genetic distance (Additional file 1: Figure S3) in LGs of Jatropha genomes. The average LD observed in this study (*r*^2^ = 0.540) was little higher than the previous study (*r*^2^ = 0.4952) in *Jatropha* [38], and lower than in chickpea (*r*^2^ = 0.59–0.62) [15].

### QTL mapping

**Fig. 2:**
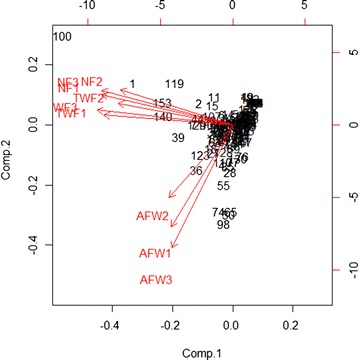
A principal coordinate analysis (PCoA) of yield traits for the *Jatropha* HY population

Fruit yield in *J. curcas* is one of the most important agronomic traits. However, selective breeding for higher yield remains the most challenging task for *J. curcas* at present. Yield traits were measured in the HY QTL mapping population, and the frequency distributions of all the traits in the progeny showed a continuous distribution. The largest NF-1, NF-2 and NF-3 values were 268, 239 and 194.3, respectively, while the smallest values were 1.0, 2.0 and 2.0, respectively, and the average values were 23.4, 24.9 and 25.9, respectively. The highest TWF-1, TWF-2 and TWF-3 values were 745, 806.5 and 602.5 g, respectively, while the lowest were 1.0, 1.5 and 1.0 g, respectively, and the average values were 86.1, 84.6 and 80.4 g, respectively. The highest AFW-1, AFW-2 and AFW-3 values were 10.4, 10.9 and 15.2 g, respectively, while the lowest were 0.3, 0.3 and 0.5 g, respectively, and the average values were 2.8, 3.2 and 3 g, respectively. As expected, all the fruit-yield traits correlated with each other, and NF had high correlation with TWF (Fig. 2).

**Fig. 3:**
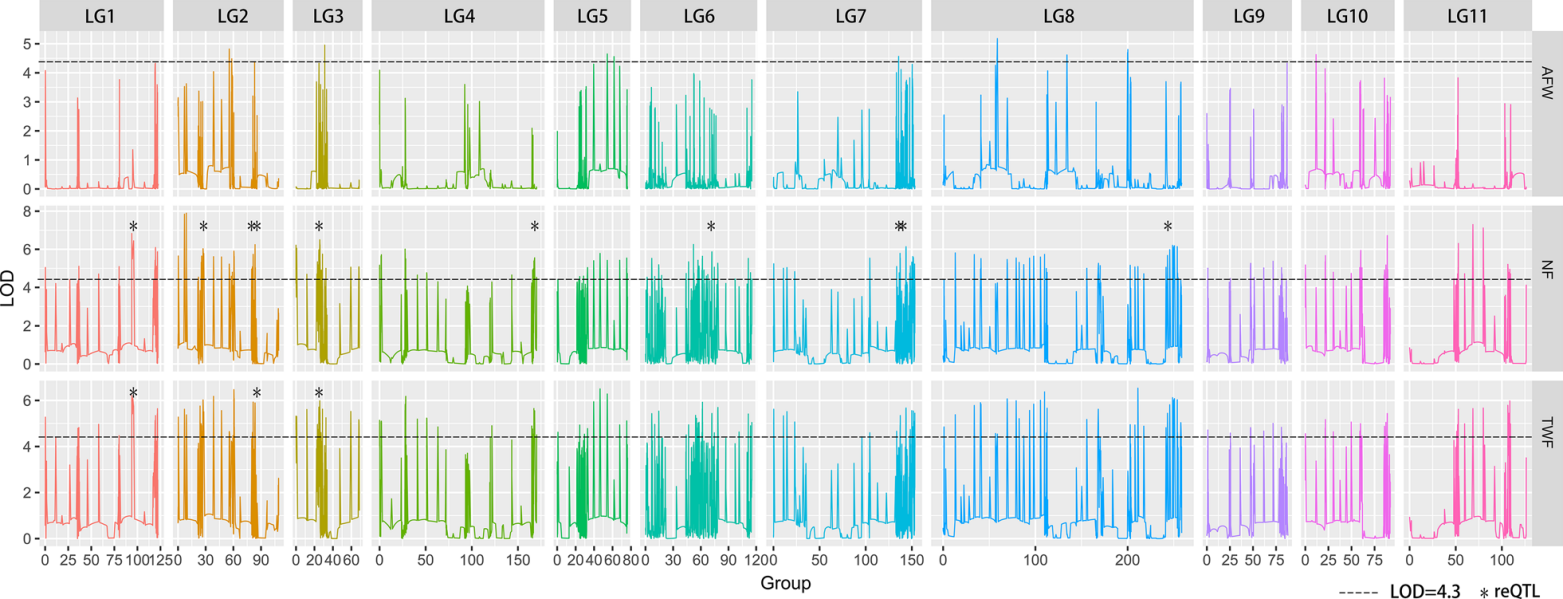
QTL mapping results. QTL mapping profiles for three yield traits, AFW (average fruit weight per fruit trait), NF (fruit number) and TWF (total fruit weight), with an LOD threshold of 4.3. The reQTLs were indicated by asterisks. Each of them were repeatable in three replicates and average

QTL analyses were performed on each of the fruit-yield traits, including NF, TWF and AFW. Thirteen reQTLs (ten for NF, three for TWF) were identified using the AFSM linkage map with an LOD threshold of 4.3, determined by permutations, and 2 LOD-support intervals that extended from 0.03 to 2.05 cM in length (Table 2, Fig. 3). The LOD score values ranged from 4.34 to 6.733. The QTL *qNF*-*1* was detected at the end of LG1 and accounted for 18.967% of the phenotypic variance in NF. At the same position, *qTWF*-*1* was detected. Two QTL pairs were also detected at the same position (*qNF*-*2b* and *qTWF*-*2* at the end of LG2, and *qNF*-*3* and *qTWF*-*3* in the middle of LG 3). The percentage of phenotypic variation explained by each reQTL ranged from 16.067% (*qTWF*-*3*) to 20.967% (*qNF*-*7b*). These reQTLs should be considered as major QTLs [39]. Interestingly, in this study, we only found reQTLs associated with the number and the total weight of fruits, but no reQTL was associated with average fruit weight per fruit, which may indicate that these reQTLs affect the fruit yield by the number of fruits, and consequently the total fruit weight, rather than the average fruit weight per fruit.

**Table 2:** Repeatable QTLs mapped for yield traits in *Jatropha*

QTL name	Trait	LG^a^	Position (cM)	Flanking markers	99% CI (cM)^d^	LOD	*R*^2^ (%)^e^	Effect	Estimatedinterval size (cM)
qNF-1	NF^b^	1	94.67	KK914295.1_173222 Jatropha666	93.88–95.93	5.273	18.967	Add	2.05
qNF-2a	NF	2	27.92	KK914286.1_1650230 Jatropha580	26.95–27.92	6.567	19.367	Add	0.97
qNF-2b	NF	2	81.51	KK914708.1_14953 Jatropha3259	81.4–81.55	6.733	19.333	Add	0.15
qNF-2c	NF	2	83.5	KK914399.1_1189397 Jatropha1302	83.23–83.58	5.367	18	Add	0.35
qNF-3	NF	3	25.76	KK914370.1_355263 Jatropha1134	25.58–26.05	5.52	18.033	Add	0.47
qNF-4	NF	4	168.11	KK915534.1_3177 Jatropha5670	167.29–168.11	5.093	18.033	Rec	0.82
qNF-6	NF	6	71.94	KK914342.1_53519 Jatropha915	71.85–72.17	5.837	18.067	Add	0.32
qNF-7a	NF	7	143.62	KK914970.1_774896 Jatropha4408	143.59–143.78	5.01	20.067	Dom	0.19
qNF-7b	NF	7	148.29	KK914352.1_426180 Jatropha984	148.29–148.31	5.11	20.967	Dom	0.02
qNF-8	NF	8	253.78	KK914383.1_100448 Jatropha1196	253.75–253.78	5.413	18.367	Dom	0.03
qTWF-1	TWF^c^	1	94.67	KK914295.1_173222 Jatropha666	93.88–93.93	4.893	19.633	Add	0.05
qTWF-2	TWF	2	81.51	KK914708.1_14953 Jatropha3259	81.4–81.51	5.257	17.4	Add	0.11
qTWF-3	TWF	3	25.76	KK914370.1_355263 Jatropha1134	25.76–25.94	4.34	16.067	Add	0.18

*Add* additive effects, *Rec* recessive effects, *Dom* dominant effects

^a^Linkage group

^b^The number of fruits

^c^The total weight of fruits (g)

^d^99% confidence interval for QTL length (cM)

^e^Proportion of phenotypic variation explained by each QTL

As complex traits, *J. curcas* yield traits are quantitative and determined by many genes with major or minor effects [12]. In this study, each of the three reQTL pairs for NF and TWF was co-localized to the same genomic regions in LG 1, 2 and 3, separately (Fig. 3). These reQTL pairs that mapped to the same locations in the genome had similar gene actions, implying that there is a genetic basis for the phenotypic correlation between NF and TWF traits, which is consistent with the high correlation observed between the two traits in phenotypic analyses. These three reQTL pairs associated with both traits, revealing that these are three critical regions for *J. curcas* fruit yield. Similarly, previous studies reported that two QTL clusters played pleiotropic roles in regulating *J. curcas* growth and seed yield, such as plant height, stem diameter, branch number, seed yield, and fruit number [12], and a major QTL plays pleiotropic roles in regulating rice heading date and yield [40]. However, the previously reported pleiotropic QTLs in *J. curcas* had long genetic distances from flanking markers because of the limited number of molecular markers [12]. Therefore, we constructed an ultrahigh-density genetic linkage map, which will lay a solid foundation for a variety of future genetic and genomic studies. Marker-assisted selection (MAS), using the closely linked markers identified in this study, can speed up the genetic improvement of *J. curcas*. The three LG regions were associated with two traits, indicating either linkage or pleiotropic effects. Although QTL studies cannot entirely distinguish between tight linkage and strict pleiotropy [41], the high resolution of QTL positions due to the dense sequence-based genome map suggests that genes with pleiotropic effects may account for the genetic variation of these correlated traits. Besides, there could be certain genes co-existing in these reQTLs or a certain gene with pleiotropic effects on *J. curcas* fruit-yield traits.

**Fig. 4:**
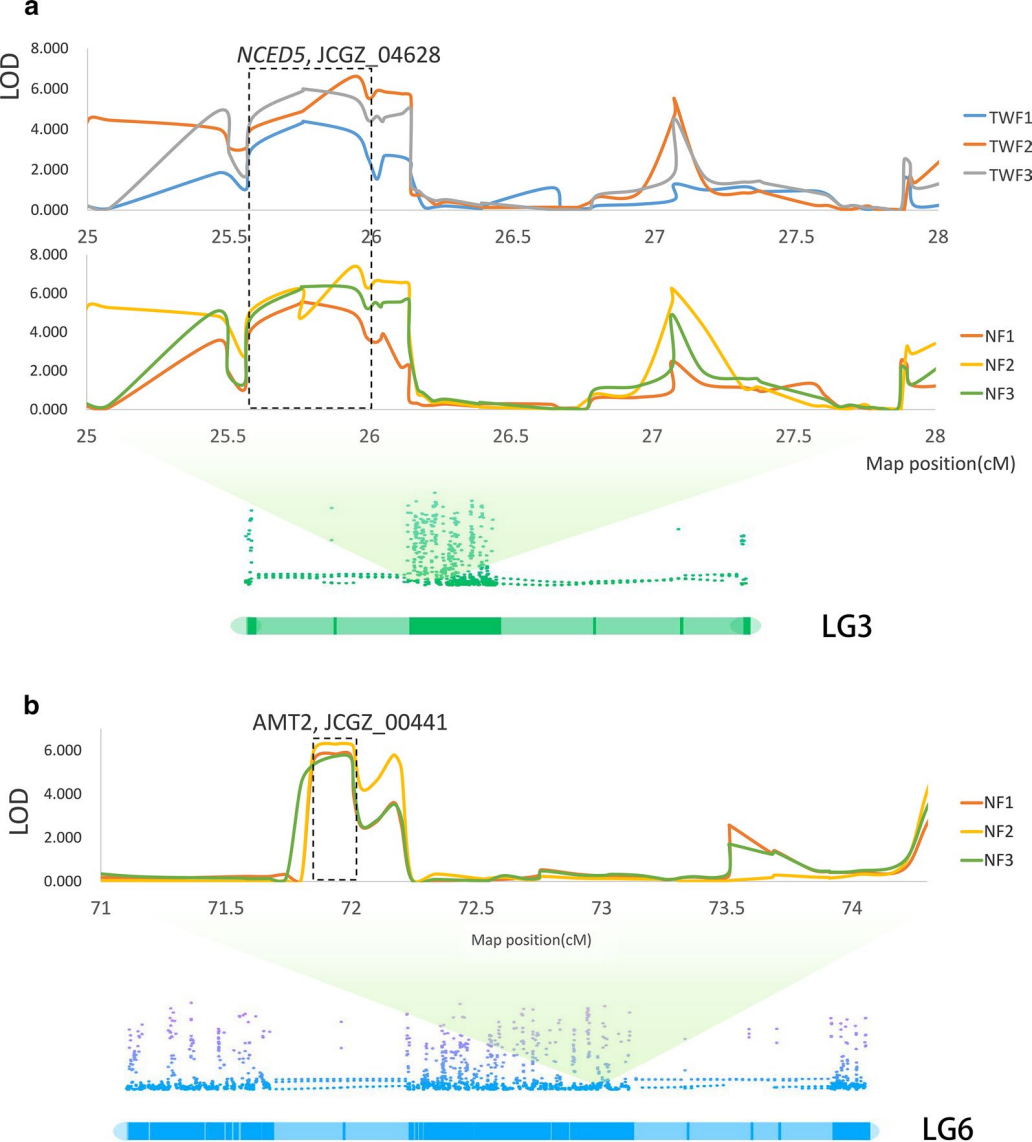
Candidate QTL genes in LG3 and LG6 for TWF and NF traits. **a** Candidate QTL gene NCED5. **b** Candidate QTL gene AMT2

### QTL candidate gene analysis

The likelihood of candidate genes corresponding to QTLs on LGs is very high, since the length of the QTL–position confidence intervals are extremely narrow. We did not expect to be able to identify all candidate genes that influence the QTLs underlying the fruit-yield traits in *J. curcas*. Nevertheless, *nine*-*cis*-*epoxycarotenoid dioxygenase 5* (*NCED5*, JCGZ_04628), which encodes 9-*cis*-epoxycarotenoid dioxygenase, a key enzyme in the biosynthesis of abscisic acid (ABA), was linked to the NF and TWF-related QTLs on LG 3 (Fig. 4a). Previous studies already showed that plant hormones play an important role in regulating flower development, especially for the female flower. It may cause a substantial increase in the female-to-male flower ratio; therefore, resulting in a greater number of female flowers and increase in number of fruits set as well as higher yield [42–46]. Wang et al. reported the ABA effects on wheat floret development and grain set, which include an inhibition of floret development and a decrease in the number of fertile florets and grain set at floret initiation, terminal spikelet formation, meiosis and floret degeneration developmental stages [47]. Zhu et al. noted that *NCED5* could influence rice inferior and superior spikelet development by regulating the expression of starch synthesis genes [48]. Further studies are still needed to expand the findings from this study to determine the relationships among the ABA signalling pathway in *J. curcas*, the developmental regulatory networks, and yield traits.

*Ammonium transporter 2* (*AMT2*, JCGZ_00441) was linked to the NF-related QTL on LG6 (Fig. 4b). As previous studies report, *AMT2*, which has ammonium transmembrane transporter activity and high-affinity secondary active ammonium transmembrane transporter activity, encodes a high-affinity ammonium transporter, expressed in roots and shoots under nitrogen and carbon dioxide regulation, respectively [49]. Ammonium is an important nutrient and a ubiquitous intermediate in nitrogen metabolism, and plants can control ammonium fluxes by regulating expression of AMT2/Rh proteins [49]. AMT/Rh-mediated ammonium transport is critical for providing sufficient nitrogen to plants for optimal growth [49] and may control the yield. Although there is an indication that this candidate gene is correlated with yield traits, further studies should be conducted to validate and prove the real function and effects of *AMT2* in *J. curcas* on gene expression in photosynthesis and nitrogen metabolism-synthesis pathways and yield traits.

## Conclusion

Our understanding of the genetic architecture of traits in *J. curcas* is increasing with the development of new analytical methods. In this study, we constructed an ultrahigh-density genetic linkage map, containing 3422 SNP and indel markers. This linkage map can provide better marker–trait associations in future studies because of its high map density. Thirteen yield reQTLs and two candidate QTL genes were identified based on this linkage map, which proved the validity and practicability of the *J. curcas* genetic map. This genetic map will also be a useful tool for the localization of other economically important QTLs and candidate genes for *J. curcas* marker-assisted selection. In addition, this genetic linkage map can be merged with other *J. curcas* genetic linkage maps into a composite map and improve the *J. curcas* reference genome sequence. Besides, this genetic linkage map can be compared with the genomes of phylogenetically related species to assess the relationships among the genomes of related species. Furthermore, the preliminary verification of possible candidate genes underlying mapped QTLs demonstrated the importance of new insights into the complex relationship between phenotypes and genotypes. QTL mapping with ultrahigh marker densities must be considered as a major step in understanding regions that control QTLs and in verifying the allelic expression of phenotypic traits in the future.

## Methods

### Plant material

An outcrossing F1 *J. curcas* mapping population (JoinMap CP population type) comprising 153 genotypes was produced between two parental lines, YN049X and HN001-31-1, and is referred to here as the HY population. HN001-31-1 is a high-yield landrace from Hainan Island, in the south of China, where it is isolated from other parts of the mainland, and YN049X is a high-yield landrace from Yunnan Province, located far from the Hainan Province. We got the hybrids between YN049X and HN001-31-1 by artificial pollination. In the flowering time, the selected female flowers were bagged to prevent from insect pollination and then marked them. Stem cuttings of 153 F1 progeny and parents were planted in Chengmai City, Hainan Province, China, using a randomized block design and two replicates, with each line containing six individuals. The population and parental lines were planted under standard growth conditions with 2 m × 2 m spacing. Three individuals from each line were harvested in 2012 for the collection of phenotypic data. We harvested the fruits of each plant in separate nylon mesh bags and dried them. The HY mapping population was evaluated for three component traits, including the total number of fruits (NF), the total weight of fruits (TWF) for each accession and the average fruit weight per fruit (AFW), with three replicates per measurement. The yield traits NF, TWF and AFW were from the total yield in the year. Each of them had three replicates (NF1, NF2, NF3, TWF1, TWF2, TWF3, AFW1, AFW2 and AFW3). Principal coordinate analysis (PCoA) was calculated among all the traits, based on the binary character matrix [50]. DNA from young leaf samples was extracted using Plant DNeasy Maxi Kit (QIAGEN, Valencia, CA) and checked on agarose gel to ensure the samples were not degraded or contaminated with ribosomal RNA. DNA concentration was assessed using the NanoDrop ND-1000 Spectrophotometer.

### AFSM library construction and sequencing

Using all 153 F1 individuals from the HY population, we constructed two sets of *J. curcas* AFSM libraries, using a previously described protocol by Xia et al. [21]. The libraries were sequenced on an Illumina HiSeq 2500 sequencing platform (Illumina, San Diego, California, USA) with 150-bp paired-end lengths. The sequence dataset was submitted to the NCBI Sequence Read Archive (SRA) under Accession numbers SRR5351047, SRR5351046 and SRR5351045.

### AFSM sequence analyses and genotyping

Raw Illumina sequence reads were checked for quality using FastQC version 0.10.1 (S. Andrews: http://www.bioinformatics.babraham.ac.uk/projects/fastqc). Custom Perl scripts [21] were used to analyse the AFSM data, confirm the barcodes and restriction sites, and filter the data. Then Bowtie2 [51] was used to align the GCA_000696525.1_JatCur_1.0_genomic *J. curcas* genome [10]. Next, SAMtools and VCFtools_v0.1.9 (http://vcftools.sourceforge./net/) were implemented in inferring the AFSM loci, call SNPs and indels at each locus, and determine genotypes.

### Linkage map construction

AFSM SNPs and indels were used for linkage map construction (CP mapping) using JoinMap 4.1 [52]. To identify these markers, all pairs of tags were evaluated for the presence of at least two reads. Markers were identified and scored by querying the filtered tags for pairs of sequences, which passed a Fisher’s exact test for independence, and fit to the expected Mendelian segregation ratio as demonstrated by a Chi-squared test (*P* < 0.01). The markers segregating for only one of the parents were scored as lm × ll (marker in female parent) or nn × np (marker in male parent), while markers segregating for both parents with two alleles were scored as hk × hk (marker in both parents). Markers most likely scored as heterozygotes due to sequencing errors were excluded and classified as missing data. Markers with more than 30% missing data were removed from the analysis. Only markers that met the above criteria were grouped using a minimum LOD (logarithm of odds) threshold of 4.0 with the Kosambi mapping function; the others were excluded. The SNP and indel markers were integrated into 11 LGs in intra-specific genetic maps based on their genetic distance in centimorgans (cM).

### Determination of linkage disequilibrium and recombination rate

We used the sliding window approach of TASSEL v5.0 to determine genome-wide LD in *Jatropha,* with the LD estimates (significant *P* value < 0.01) as average squared-allele frequency correlations (*r*^2^) among marker pairs which mapped on genetic map. The decay of LD with the genetic distance was measured by the *r*^2^ values of marker pairs across 11 LGs of genetic map. The graph was plotted between pooled *r*^2^ and genetic distance (cM) based on nonlinear regression model considering the *r*^2^ value = 1 at marker genetic distance of 0 cM to determine the trend of LD decay in *Jatropha* genomes. Recombination rate analysis was evaluated using FastEPRR with the nonoverlapped sliding window length of 5 cM [53].

### QTL and candidate gene analysis

QTL calculations were completed using the software MapQTL5 [54]. rMQM (Restricted MQM Mapping) was used to map QTLs and estimate their effects. The logarithmic (LOD) score of significant QTLs was determined by conducting test analyses (1000 permutations, 5% total error level). Since many adjacent markers in the AFSM linkage map had a map distance of < 1 cM, the confidence intervals of the QTL are as described previously [12, 55]. In short, the maximum LOD scores were used as the QTL positions, and LOD scores were within the maximum 2-LOD-unit confidence intervals. The percentage of contribution (PVE) of each identified QTL to the total phenotypic variance was estimated by variance analysis. The QTL names start with “*q*.”

To identify candidate genes underlying the QTLs, we used a BLASTN search of the AFSM SNP and indel markers mapped to the QTL regions followed by a BLASTX search against the NCBI non-redundant protein database.

## Additional file

**Additional file 1: Figure S1.** Recombination hotspots in 11 LGs. **Figure S2.** Distribution of r^2^ values between markers for *Jatropha*. **Figure S3.** Genome-wide LD decay (mean r^2^) estimated using 3422 markers that are mapped on 11 LGs of *Jatropha* ultra-high density genetic map. **Table S1.** Recombination hotspots in 11 LGs. **Table S2.** Genome-wide LD estimates among marker-pairs mapped across 11 LGs of ultra-high density genetic map for *Jatropha*.
