# Supplementary material for: Construction of an ultrahigh-density genetic linkage map for Jatropha curcas L. and identification of QTL for fruit yield
Source: Biotechnol Biofuels. 2018 Jan 9;11:3. doi: 10.1186/s13068-017-1004-9 (PMC5759280; doi:10.1186/s13068-017-1004-9)
Supplement: Supplementary file 1 — Additional file 1: Figure S1. Recombination hotspots in 11 LGs. Figure S2. Distribution of r2 values between markers for Jatropha. Figure S3. Genome-wide LD decay (mean r2) estimated using 3422 markers that are mapped on 11 LGs of Jatropha ultra-high density genetic map. Table S1. Recombination hotspots in 11 LGs. Table S2. Genome-wide LD estimates among marker-pairs mapped across 11 LGs of ultra-high density genetic map for Jatropha. [file 13068_2017_1004_MOESM1_ESM.pdf]

Construction of an ultrahigh-density genetic linkage map for *Jatropha curcas* L. and identification of QTL for fruit yield

Zhiqiang Xia, Shengkui Zhang, Mingfu Wen, Cheng Lu, Yufang Sun, Meiling Zou and Wenquan Wang

Additional File 1	Title
Figure S1	Recombination hotspots in 11 LGs
Figure S2	Distribution of r^2 values between markers for Jatropha.
Figure S3	Genome-wide LD decay (mean r^2) estimated using 3422 markers that are mapped on 11 LGs of *Jatropha* ultrahigh-density genetic map.
Table S1	Recombination hotspots in 11 LGs.
Table S2	Genome-wide LD estimates among marker-pairs mapped across 11 LGs of ultrahigh-density genetic map for *Jatropha*.

Figure S1 Recombination hotspots in 11 LGs.

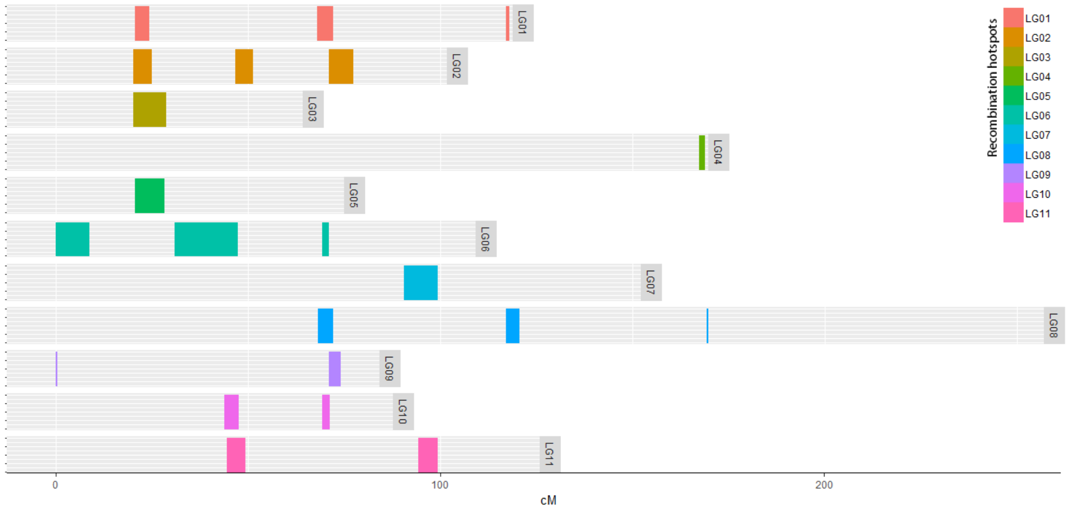

Figure S2 Distribution of r^2 values between markers for Jatropha.

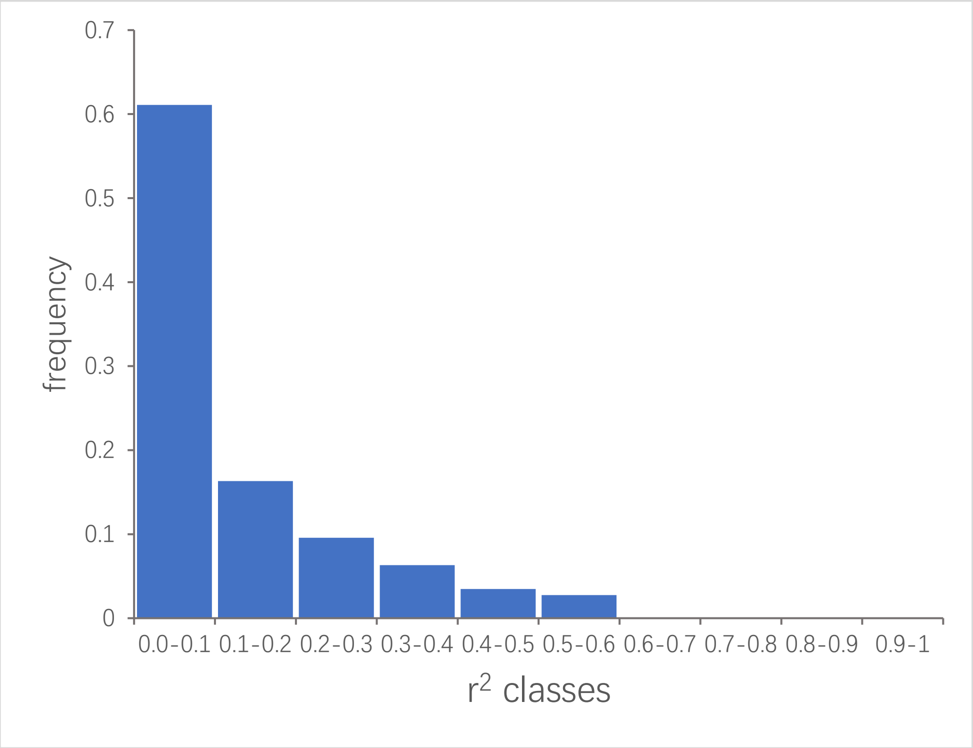

Figure S3 Genome-wide LD decay (mean r^2) estimated using 3422 markers that are mapped on 11 LGs of *Jatropha* ultrahigh-density genetic map. The r^2 value for the marker genetic distance of 0 cM is defined as 1. The dots indicate the mean r^2 values. The curve was drawn across the dots using the nonlinear regression model.

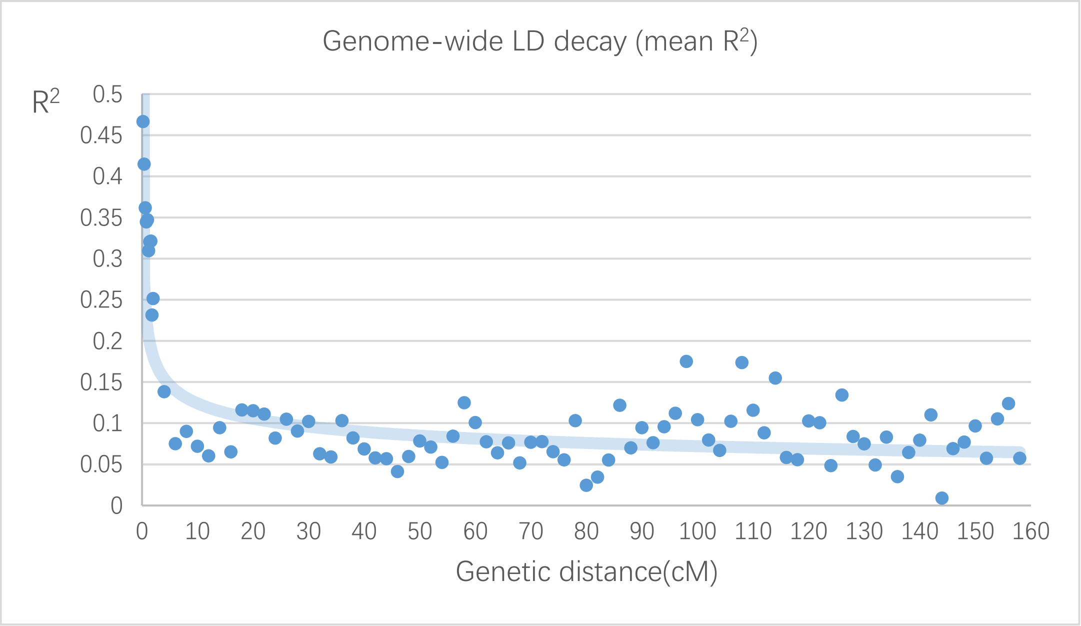

Table S1 Recombination hotspots in 11 LGs.

	LG length (cM)	No.	Start-end (cM)
**LG1**	122.51	3	44.42-62, 146.62-165.2, 252.82-264.26
**LG2**	109.10	3	43.62-63.6, 101.02-120.2, 153.62-176.6
**LG3**	68.67	1	43.42-71.4
**LG4**	170.31	1	361.22-373.98
**LG5**	76.20	1	44.42-70.6
**LG6**	115.35	3	0.16-28.14, 66.56-111.54, 149.56-163.04
**LG7**	153.40	1	195.82-224.1
**LG8**	258.60	3	147.1-165.48, 253.1-269.88, 365.9-376.04
**LG9**	87.86	2	0.02-10.2, 153.22-169.28
**LG10**	92.13	2	94.62-112.2, 149.42-163.22
**LG11**	126.45	2	96.28-115.86, 203.48-223.86
**Total**	1380.58	22	/

Table S2 Genome-wide LD estimates among marker-pairs mapped across 11 LGs of ultrahigh-density genetic map for *Jatropha*.

Linkage Groups (LGs)	Number of marker-pairs used	* Marker-pairs in significant LD	LD-estimates (average r^2)*
LG1	97,188	3,539 (3.641%)	0.489
LG 2	230,252	6,049 (2.627%)	0.539
LG 3	157,672	2,549 (1.617%)	0.545
LG 4	71,059	2,077 (2.923%)	0.544
LG 5	163,582	6,435 (3.934%)	0.784
LG 6	390,154	10,912 (2.797%)	0.526
LG 7	224,798	8,863 (3.943%)	0.564
LG 8	201,624	4,797 (2.379%)	0.459
LG 9	128,210	3,557 (2.774%)	0.474
LG 10	151,094	3,888 (2.573%)	0.478
LG 11	159,543	4,830 (3.027%)	0.538
Total LGs	1,975,176	57,496 (2.911%)	0.540

*Marker-pairs at significant ($p<0.01$) LD
